# Propolis Extract for Onychomycosis Topical Treatment: From Bench to Clinic

**DOI:** 10.3389/fmicb.2018.00779

**Published:** 2018-04-25

**Authors:** Flavia F. Veiga, Marina C. Gadelha, Marielen R. T. da Silva, Maiara I. Costa, Brenda Kischkel, Lidiane V. de Castro-Hoshino, Francielle Sato, Mauro L. Baesso, Morgana F. Voidaleski, Vanessa Vasconcellos-Pontello, Vânia A. Vicente, Marcos L. Bruschi, Melyssa Negri, Terezinha I. E. Svidzinski

**Affiliations:** ^1^Laboratório de Micologia Médica, Departamento de Análises Clínicas e Biomedicina, Universidade Estadual de Maringá, Maringá, Brazil; ^2^Programa de Pós-Graduação em Física, Departamento de Física, Universidade Estadual de Maringá, Maringá, Brazil; ^3^Microbiology, Parasitology and Pathology Post-Graduation Program, Department of Pathology, Federal University of Paraná, Curitiba, Brazil; ^4^Laboratory of Research and Development of Drug Delivery Systems, Department of Pharmacy, Universidade Estadual de Maringá, Maringá, Brazil

**Keywords:** dermatophytosis, natural products, propolis, antifungal activity, *ex vivo* nail model, photoacoustic spectroscopy, permeation property

## Abstract

Onychomycosis is a chronic fungal infection of nails, commonly caused by dermatophyte fungi, primarily species of *Trichophyton*. Because of the limited drug arsenal available to treat general fungal infections and the frequent failure of onychomycosis treatment, the search for new therapeutic sources is essential, and topical treatment with natural products for onychomycosis has been encouraged. Propolis, an adhesive resinous compound produced by honeybees (*Apis mellifera*), has shown multiple biological properties including significant antifungal and anti-biofilm activities *in vitro*. In spite of promising *in vitro* results, *in vivo* results have not been reported so far. This study assessed an ethanol propolis extract (PE) as a topical therapeutic option for onychomycosis, including its characterization *in vitro* and its applicability as a treatment for onychomycosis (from bench to clinic). The *in vitro* evaluation included analysis of the cytotoxicity and the antifungal activity against the planktonic cells and biofilm formed by *Trichophyton* spp. We also evaluated the capacity of PE to penetrate human nails. Patients with onychomycosis received topical PE treatments, with a 6-month follow-up period. The results of the *in vitro* assays showed that PE was non-toxic to the cell lines tested, and efficient against both the planktonic cells and the biofilm formed by *Trichophyton* spp. The results also showed that PE is able to penetrate the human nail. The results for PE applied topically to treat onychomycosis were promising, with complete mycological and clinical cure of onychomycosis in 56.25% of the patients. PE is an inexpensive commercially available option, easy to obtain and monitor. Our results indicated that PE is a promising natural compound for onychomycosis treatment, due to its ability to penetrate the nail without cytotoxicity, and its good antifungal performance against species such as *Trichophyton* spp. that are resistant to conventional antifungals, both *in vitro* and in patients.

## Introduction

Onychomycosis, part of the dermatomycosis group, is a chronic fungal infection of the nails. This kind of mycosis is a frequent disorder that causes approximately 50% of all human nail diseases, although in the literature this prevalence seems to be underestimated (Sigurgeirsson and Baran, 2014).

Several investigators have suggested that the incidence of onychomycosis has increased over the last years (Sigurgeirsson and Baran, 2014; Gupta et al., 2016; Maraki and Mavromanolaki, 2016; Bodman and Krishnamurthy, 2017). However, its prevalence varies according to the location and population. A few recent studies, with populations of all ages, show prevalences between 1 and 8% in the United States (Bodman and Krishnamurthy, 2017), 14% in Italy (Papini et al., 2015), and 16% in Greece (Maraki and Mavromanolaki, 2016). In elderly people, epidemiological studies have found prevalences of around 35% in Italy (Cozzani et al., 2016), between 9.8 and 22.4% in Poland (Reszke et al., 2015), and 46.5% in Brazil (Chiacchio et al., 2013). Therefore, onychomycosis is significantly higher in the elderly population than in children and younger adults (Maraki and Mavromanolaki, 2016; Dubljanin et al., 2017).

Onychomycosis is also known as tinea unguium because it is caused mainly by dermatophyte fungi, followed by yeasts and non-dermatophyte environmental factors. Among these agents, the dermatophytes are the most frequent, following by yeasts, especially *Candida* spp. and non-dermatophyte molds (NDM) (Baswan et al., 2017; Bodman and Krishnamurthy, 2017).

Treatment of onychomycosis remains a challenge to patients and professionals, because of the difficulty in attaining a definitive cure and the high recurrence rate (Westerberg and Voyack, 2014). Systemic antifungal therapy is restricted to limited options including azoles (itraconazole and fluconazole), terbinafine (LaSenna and Tosti, 2015), and griseofulvine (Kreijkamp-Kaspers et al., 2017). The use of systemic antifungals is associated with significant side effects and interaction with other drugs. Terbinafine is the preferred drug for oral treatment of dermatophytosis, because it inhibits all genera of dermatophytes; however, there are reports of clinical resistance to the drug and acute hepatic toxicity (LaSenna and Tosti, 2015).

The topical route of administration apparently is preferred over the systemic. However, a few researchers stand by the idea that topical medication would be more advantageous to treat onychomycosis. The reason is that the medication is applied directly on the affected area, minimizing possible interactions with other drugs (Akhtar et al., 2016). Topical application is recommended especially for mild-to-moderate nail involvement (Gupta et al., 2017).

A few drugs, e.g., amorolfine and ciclopirox are currently available for topical treatment. However, these medications also have limitations since both of them cause side effects such as a burning sensation, itching, irritation, and redness (Westerberg and Voyack, 2014).

Antifungals available for the treatment of onychomycosis show a range of adverse effects that may cause patients to stop the treatment due to the comorbidities that are usually related to this disorder. The few existing studies indicate that these natural compounds are a promising alternative for treatment of onychomycosis (Halteh et al., 2016).

Propolis is a gomo-resinous compound produced by honeybees (*Apis mellifera* L.) by mixing salivary secretions and beeswax with exudate gathered from plants (Oryan et al., 2018). Bees use this material to seal open spaces in the hives and to protect the hive from different pathogenic agents (Marcucci et al., 2001). Among the several components of propolis are phenolic substances, including flavonoids, a group of metabolites with antifungal potential (Agüero et al., 2014; Firdaus et al., 2016). Propolis has several medicinal properties including anti-inflammatory (Cavendish et al., 2015). Recently, Alves de Lima et al. (2018) demonstrated that propolis has an important role in the *Candida-*host relationship.

The main fraction of propolis is the ethanol extract, which has shown *in vitro* activity against different species of human pathogenic fungi, including *Candida albicans, Candida tropicalis, Candida glabrata, Candida parapsilosis, Trichosporon* sp., *Rhodotorula* sp., *Trichophyton* sp., and *Microsporum* sp. (Agüero et al., 2014; Firdaus et al., 2016). Some investigators have demonstrated the antifungal action of PE *in vivo*, suppressing animal sporotrichosis caused by *Sporothrix brasiliensis*, even showing an effect on isolates that were resistant to itraconazole (Silici and Koc, 2006; Waller et al., 2017).

The combination of these properties makes it possible to consider propolis as a candidate for therapeutic use as a topical antifungal. Although *in vitro* results are promising, no studies have yet demonstrated the applicability of these properties in patients with onychomycosis. The present study assessed PE as a topical therapeutic option to treat onychomycosis, from its characterization *in vitro* to its applicability *in vivo*.

## Materials and Methods

### Propolis Origin and Ethanol Extract Characterization

The sample of green propolis used in this study was collected from hives located in northern Paraná state, Brazil. The apiary is surrounded by native forest, mainly *Baccharis dracunculifolia* and eucalyptus.

For *in vitro* and *ex vivo* assays, an ethanol propolis extract (PE) was prepared with a propolis/ethanol ratio of 30/70 (w/w) by turbo extraction, centrifuged at 3500 rpm three times for 15 min each, with two 5 min intervals. PE was filtered through filter paper and filled to the initial weight with ethanol (Bruschi et al., 2003). PE was characterized for relative density, pH, dryness residue (DR), and total phenol content (TPC) (Teixeira et al., 2010; Dota et al., 2011).

The DR of the extract was determined by using exactly 10 g of PE, which was concentrated in a water bath (100°C), with occasional shaking, and dried on the infrared analytical balance (Gehaka, São Paulo, Brazil) at 110°C; the final weight was designated the DR value. At least three replicates were carried out to estimate the inherent variability of each determination. The TPC of each extract was analyzed by the Folin–Ciocalteu method as described by Teixeira et al. (2010), using a validated calibration curve with solutions of gallic acid as reference (Pereira et al., 2013). TPC was expressed as the percentage by weight of total phenolic substances in the extractive solution and corresponds to the mean of six replicates.

For treatment of selected patients we used 10% PE, available commercially in extract form from the Teaching Pharmacy of the Universidade Estadual de Maringá.

### *In Vitro* Effect of Ethanol Propolis Extract

#### Cytotoxicity Assay

For the cytotoxicity experiments, HeLa and Vero cells were cultured at 37°C in 5% CO_2_ in Dulbecco’s modified Eagle’s medium (DMEM; Sigma–Aldrich, St. Louis, MO, United States) containing 10% fetal bovine serum (FBS; Life Technologies, Itapevi, Brazil) and 1% penicillin-streptomycin (Pen Strep; Gibco, Grand Island, NY, United States). After reaching 80% confluence, the cells were detached using 25% trypsin-ethylenediaminetetraacetic acid solution (EDTA; Gibco, Grand Island, NY, USA). The cell concentration was adjusted to 2 × 10^5^ cells/mL with fresh DMEM, and the suspension was added to the wells of a 96-well plate and incubated for 24 h.

Prior to the cytotoxicity assays, the wells were washed twice with PBS. PE at 10 serial concentrations ranging from 0.001 to 0.71% concentration of TPC diluted in DMEM were added to the cells and incubated overnight at 37°C under 5% CO_2_. Cells treated with the corresponding percentage of ethanol were used as a control. Afterward, cytotoxicity with PE was assessed using the MTT assay (bromide [3-(4,5-dimethyl-thiazol-2-yl)-2,5-diphenyltetrazolium bromide]; Invitrogen, Oregon, United States) following the manufacturer’s instructions. A control was performed by measuring the cellular activity of human cells grown under the same conditions but in the absence of PE. The cytotoxicity of the PE was presented as the average of three independent experiments with three replicates.

#### Fungal Strains

The *in vitro* effect of PE on fungal cells was assessed with 45 isolates from patients with onychomycosis, including 29 *Trichophyton rubrum*, 13 *Trichophyton mentagrophytes*, 2 *Trichophyton verrucosum*, and 1 *Trichophyton interdigitale.* In each experiment, the isolates were grown on Sabouraud Dextrose Agar (SDA; Difco^TM^, Detroit, United States) for 7 days at 25°C.

#### Antifungal Activity

*In vitro* antifungal susceptibility testing of planktonic cells was performed using a microdilution method adapted from Galletti et al. (2016) and Clinical and Laboratory Standards Institute [CLSI] (2008) M38-A2 protocol. For this test, the serial dilution was performed at a ratio of two, from 1:2 to 1:1024. In this way, PE’s concentrations ranged from 0.001 to 0.71 % of TPC were made in RPMI 1640 (RPMI Medium 1640; Gibco, Grand Island, NY, United States) with l-glutamine (with sodium bicarbonate) and 0.165 M 3-(N-morpholino) propanesulfonic acid (pH 7.2) as a buffer (Sigma–Aldrich, St. Louis, United States). Fungal growths from all strains were collected from 7-day-old growing colonies in solid medium at 25°C and placed in a 0.85% sterile saline solution. The suspension was filtered in sterilized glass wool, after heavy shaking to obtain the isolated conidia. The cell density was adjusted to 5 × 10^4^ conidia/mL, using a Neubauer chamber. After incubation at 37°C for 48 h, minimum inhibitory concentrations (MICs) were determined by direct observation. The results of the MIC relative to the TPC were defined as the concentration of TPC that reduced 100% of the growth compared to the organisms grown in the absence of the drug. The minimum fungicidal concentration (MFC) was estimated by plating the suspensions exposed to different PE concentrations on SDA. The plates were incubated at 37°C for 24 h. The MFC was defined as the lowest concentration of the test compound in which the microorganisms showed no recovery. These tests were performed in duplicate.

#### Anti-biofilm Activity

In order to evaluate the effect of PE on the biofilm of the different fungal species, PE was added on the plate after 24 h of biofilm formation. This assay was based on the methodology described by Galletti et al. (2016), with modifications. Clinical isolates of *T. rubrum* CMRP2912 and *T. interdigitale* CMRP2921 were used. The inoculum of each strain was adjusted to a final concentration of 1 × 10^6^ conidia/mL on RPMI 1640, and 200 μL of each suspension was placed on a 96-well plate. Negative controls (200 μL of RPMI 1640 medium alone) were also included. The plates were incubated at 37°C in a shaker at 110 rpm for 24 h.

After 24 h of biofilm formation, the medium was aspirated and the non-adherent cells were removed by washing the biofilms once with 200 μL of sterile saline. Then, 200 μL of PE (0.088% concentration of TPC in RPMI 1640 medium) was added to each well. Untreated controls (200 μL of fungal biofilm with RPMI 1640 medium) were also included. The biofilms with PE were incubated for a further 24 h, at 37°C on a shaker at 120 rpm/min. The effect of PE on the preformed biofilms was assessed by quantifying the number of CFUs and biomass as described previously (Galletti et al., 2015). All experiments were repeated on three occasions with individual samples.

### *Ex Vivo* Effect of Ethanol Propolis Extract

#### Evaluation of PE Penetration Into Nail by Photoacoustic Spectroscopy (PAS)

**FIGURE 1:**
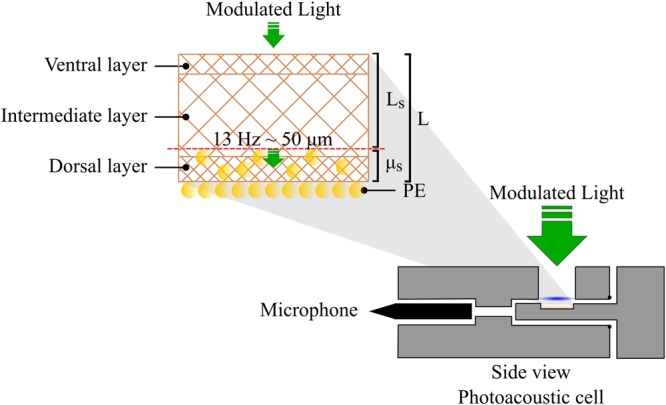
Schematic diagram of placement of nail fragment with propolis extract (PE) in the photoacoustic cell. μs = thermal diffusion length (μm); L = initial sample thickness (μm); Ls = field sample thickness (μm).

Initially, distal nail fragments were obtained from the hands of healthy volunteers, without causing any harm or pain. After the collection, these fragments were cut into equal pieces of 1 cm^2^ and 0.2 μL of PE was applied on the dorsal surface of the nail fragment. The PAS analysis was performed 24 h after the application. The measurements were obtained with a simple experimental arrangement (Ames et al., 2017; Borghi-Pangoni et al., 2017), using the photoacoustic cell illustrated in **Figure 1**. The photoacoustic spectra were obtained in the ultraviolet and visible spectrum regions, with a wavelength range of 250–800 nm, the power of the source was 800 W and the light modulation frequency was 13 Hz. In order to evaluate the PE penetration into the nail, the samples were illuminated first on the dorsal side and then on the ventral side until the optical absorption band of PE was detected by PAS. To reach all layers of the nail, its surface was polished manually with smooth movements and the reading was repeated until the PE absorption band appeared. The modulated light on the surface of the sample (nail) may reach different depths, according to the modulation frequency used. The layer of the sample in which the radiation is absorbed is converted to heat, which contributes to the photoacoustic signal, which is obtained by calculating the thermal diffusion length (μ_s_). This parameter is mathematically defined as μ_s_ *=* (*d*/π*f*)^½^, where *d* is the thermal diffusivity of the sample and *f* is the frequency of light modulation. Using *d* = 10 × 10^-4^ cm^2^ s^-1^ (Dias et al., 2007) for thermal diffusivity of the nail (dorsal and ventral), the μ_s_ ∼50 μm.

### Clinical Investigations of the Effect of Ethanol Propolis Extract

#### Selection of Patients

The study was conducted with patients with suspected onychomycosis who were seen in the Teaching and Research Laboratory of Clinical Analysis (LEPAC), division of Medical Mycology, Universidade Estadual de Maringá, between January 2015 and December 2017. The study conformed to resolution 196/1996 from the CNS-MS (National Council of Health), under the supervision of the Standing Committee on Ethics in Research Involving Human Beings, under registration numbers 615.643 (isolation and identification of microorganisms) and 44.896515.5.0000.0104 (treatment). The participants provided written and signed consent for publication of their results.

#### Mycological Culture and Fungi Identification

The nail samples from patients with clinical suspicion of onychomycosis were collected by scraping the nails after the sites were cleaned with 70% ethanol. The nail scrapings were cleared with 20% KOH plus Parker’s blue–black permanent ink (3:1) and observed by direct microscopy. The samples were also cultured on SDA and selective agar for pathogenic fungi supplemented with cycloheximide (Difco^TM^, Detroit, United States), with nine inoculations in each kind of culture medium. These cultures were maintained at 25°C for up to 30 days in order to allow any fungi present in the sample to develop, and the growth of fungi was assessed daily. The fungi were preliminarily identified according to their macromorphology and micromorphology such as colony color and texture, border type, radial growth rate, and characteristics of the hyphae, conidiophores, and conidia (de Hoog et al., 2018). The isolated and identified fungi were stored freeze-dried in the Mycology Collection of the Laboratory of Medical Mycology, Universidade Estadual de Maringá. The two strains used in the anti-biofilm assays were deposited in the Microbial Collections of Paraná Network – TAXon line at the Federal University of Paraná, under registration numbers CMRP2912 and CMRP2921. The PCR amplification and sequencing were performed on the ITS1-5.8S rDNA-ITS2 region, using the universal primers ITS1 and ITS4 (White et al., 1990) according to Vicente et al. (2008), in an ABI3730 automated sequencer (Applied Biosystems). Comparisons of ITS region were performed using GenBank and a BLAST analysis, and the CBS databank^1^. These strains were identified as *T. rubrum* (CMRP2912) and *T. interdigitale* (CMRP2921) and added to GenBank under numbers MG976820 and MG976819, respectively.

#### Treatment

Commercially available PE was provided by the Pharmacy of the Universidade Estadual de Maringá, and the treatment was monitored by a dermatologist. The criteria for inclusion in the study were: patients with a positive culture, a fungus that had been isolated and identified, who stated that they had not used any antifungals previously, and who gave their written consent for treatment of the onychomycosis only by topical application of 10% PE. The 16 participating patients were instructed to clean their nails with soap, water, and a brush daily and to polish the affected areas of the nails weekly. Thereafter, they were to apply two drops of PE on the affected area twice a day. The dermatologist monitored the treatment evolution every 2 months; the patients reported their experience, including difficulties or side effects, during that period of time, and the dermatologist answered questions, documented the evolution of the infection, and reinforced the recommendations. After 6 months of treatment, the patients were classified according to the outcome: (i) without apparent improvement (the appearance and size of the lesions remained similar to the state before the treatment began and abundant fungi were still present in the sample); (ii) partial improvement (the ungual lesion was at least 50% smaller and only sparse fungi were present in the sample); (iii) complete cure (the nail was fully recovered to its pre-infection appearance and no fungi could be observed in the sample).

### Statistical Analysis

The software OriginPro 8.0 (OriginLab Corporation, Northampton, MA, United States) was used for statistical analysis. Data were statistically evaluated by one-way analysis of variance (ANOVA) and *post hoc* comparisons of the means of individual groups were performed using Tukey’s Honestly Significant Difference test. Values of *p* < 0.05 were considered statistically significant.

## Results

### Characterization of Propolis Extract

The physical and chemical evaluation of the PE showed the following characteristics: pH = 5.12 ± 0.05 and relative density = 0.8722 ± 0.0009 g/mL. The DR value was 19.33% ± 0.01 (w/w) and the TPC value was 1.42% ± 0.07 (w/w).

### *In Vitro* Effect of Ethanol Propolis Extract

#### Cytotoxicity Evaluation

**FIGURE 2:**
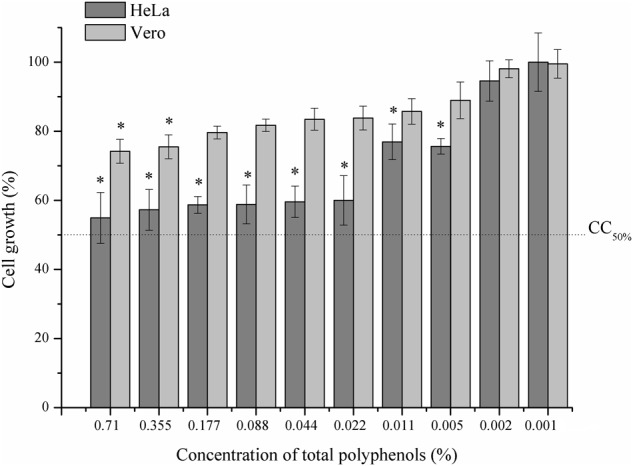
Effect of propolis extract (PE) on HeLa and Vero cell lines. Viability determined by MTT assay. Concentrations of the PE extract ranged from 0.002 to 1.42% of total polyphenols. The results are presented as the average of three independent experiments performed in three replicates, ^∗^*p* < 0.05 compared to control group.

The cytotoxicity of PE to mammal cells was assessed through an assay of MTT reduction in HeLa and Vero cell lines. From the reduction of MTT by the viable cells, it was possible to calculate the cytotoxicity index (CC_50%_), indicating the concentration of PE that would induce 50% cell lysis or death. This value was considered the threshold of acceptable cytotoxicity. As shown in **Figure 2**, the cytotoxic effect of PE on cells was dose-dependent. The HeLa cell line was more susceptible than the Vero cell line. However, considering the cytotoxicity threshold established, none of the PE concentrations tested showed a cytotoxic effect on either cell line after 24 h of exposure.

### Antifungal Activity of Propolis Extract

In all cases, the MIC was identical to the MFC and in general the values were low (**Table 1**), although the TPC varied significantly (from 0.005 to 0.355%). The majority of the isolates showed an MFC_50_ below the concentration of 0.088% TPC. For all isolates tested, the MIC and MFC values were lower than the TPC level found in the commercial PE (1.42% TPC), and therefore these fungi were considered susceptible to the medication.

**Table 1:** Evaluation of Minimum Inhibitory Concentration (MIC) and Minimum Fungicidal Concentration (MFC) of propolis extract (PE) against planktonic cells of *Trichophyton* spp. isolated from onychomycosis cases.

Strains	MIC/MFC^∗^ of PE (% Concentration of TPC)		
	Range	MIC_50_	MIC_90_
*T. rubrum* (29)	0.005–0.355	0.088	0.177
*Trichophyton* spp. (16)	0.044–0.355	0.044	0.088

*^∗^MIC/MFC: corresponded to the same values. TPC: Total phenol content in PE. MIC_*50*_ and MIC_*90*_ were defined as the MIC of PE capable of inhibiting 50 and 90% of the population of fungi evaluated, respectively.*

**FIGURE 3:**
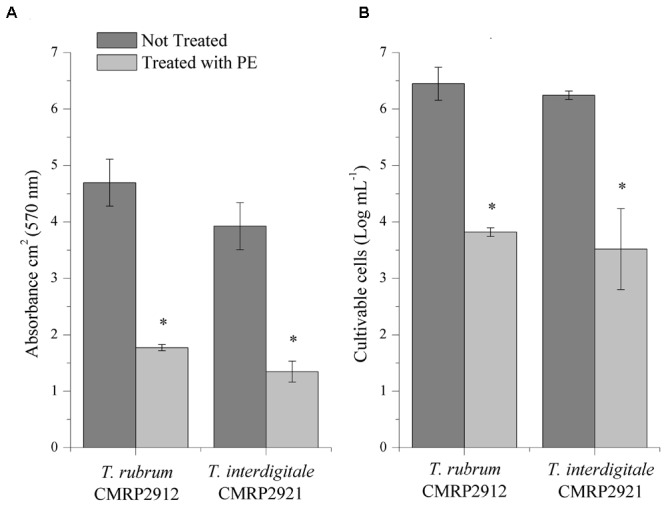
*In vitro* biofilm inhibition by propolis extract (PE) measured through two methods: Crystal violet-staining biomass production **(A)** and Colony-Forming Units **(B)** of *T. rubrum* CMRP2912 and *T. interdigitale* CMRP2921 biofilms exposed to PE. ^∗^*p* < 0.05 compared to untreated control group.

The *in vitro* effect of PE on preformed biofilms was tested against two clinical isolates of *Trichophyton* (*T. rubrum* CMRP2912 and *T. interdigitale* CMRP2921). The concentration of PE used in this study (0.088% TPC content) was based on the antifungal susceptibility test. The total biomass and the number of viable cells of the biofilms treated with PE were significantly (*p* < 0.05) lower than in the control without PE for both isolates (**Figure 3**). Therefore, PE with 0.088% TPC content was able to reduce nearly log_2_ or more of cells in the preformed *Trichophyton* biofilm.

### *Ex Vivo* Effect of Propolis Extract

**FIGURE 4:**
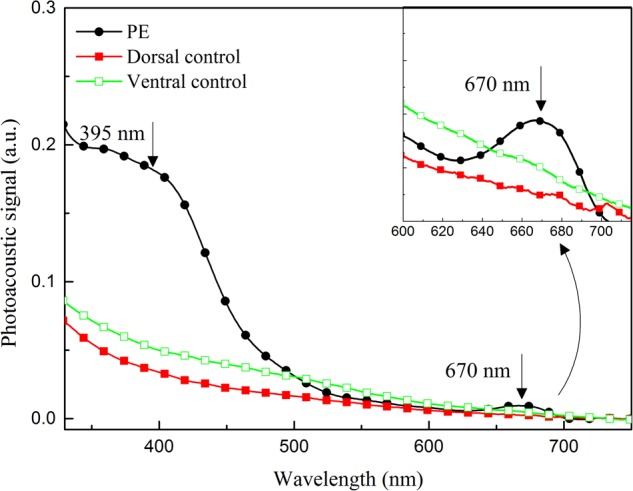
Photoacoustic spectra of: propolis extract (PE); dorsal surface of nail without PE application (Dorsal control); ventral surface of nail without PE application (Ventral control). Arrows indicate bands of optical absorption of PE. In right corner, the band centered at 670 nm.

The capacity of PE to penetrate the human nail was assessed by means of the PAS technique, in an *ex vivo* model. The isolated PE showed an optical absorption of 250–700 nm, mainly with bands around 395 and 670 nm, regions characteristic of the optical absorption of flavonoids in PE. The nail showed no optical absorption above 500 nm. Therefore, this model was considered suitable to evaluate the PE penetration through the thickness of the nail layers, because it was possible to detect the characteristic bands of each component (nail and PE), showing the typical spectra of each, and also to identify the presence of the substance applied topically, acting as a marker, in the different layers of the nail (**Figure 4**).

**FIGURE 5:**
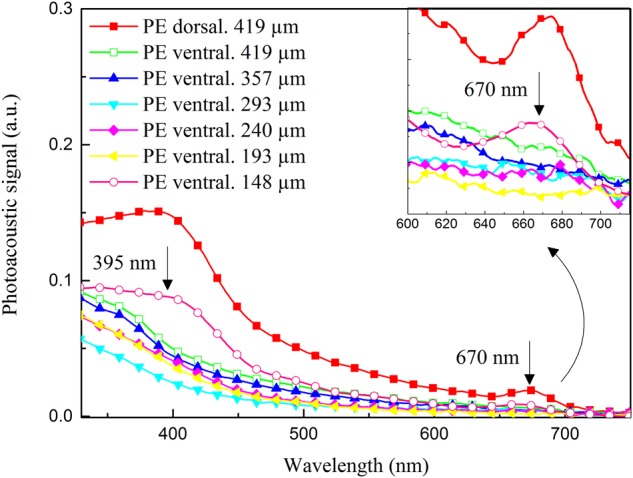
Photoacoustic spectra of nail sample after 24 h of treatment with propolis extract (PE), for different thicknesses. Surface of application of PE (PE dorsal, 419 μm); first measurement without filing the layer on the side opposite to the application site (PE ventral. 419 μm); consecutive measurements after filing the nail layer on the side opposite to the application site until optical absorption bands of PE were detected (PE ventral. 357 μm; PE ventral. 293 μm; PE ventral. 240 μm; PE ventral. 193 μm; PE ventral. 148 μm). Arrows indicate the presence of characteristic bands of PE in 148-μm-thick nail layer, highlighting the band centered at 670 nm.

Considering the thermal-diffusion length of the nail, the results of the evaluation of the PE penetration profile 24 h after it was applied to the nail fragment suggest that the extract extended around 100 μm deeper in the nail than the site where it was applied. This was clearly demonstrated by the increase in the optical-absorption bands centered at approximately 395 and 670 nm, characteristic of the PE spectrum, in the 148-μm-thick sample of nail (**Figure 5**).

### Clinical Investigations of the Effect of Propolis Extract

**FIGURE 6:**
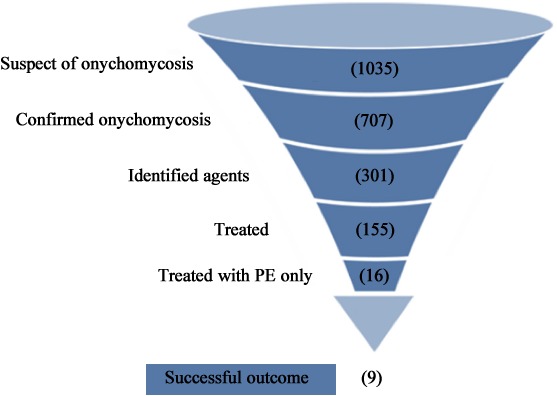
Flow chart of the experimental scheme of the study, from the number (in parentheses) of onychomycosis cases investigated to the successful outcome of treatment with PE. Treated patients, but in other health services where they received other medications and therefore were excluded from this project step. Treated with PE: number of patients who agreed to use PE as the only antifungal drug. Successful outcome: complete recovery of the anatomical appearance of the nail and absence of fungi in the sample.

The clinical evaluation of the efficacy of 10% PE in treating onychomycosis was conducted with 1035 patients with suspicion of onychomycosis who were referred to the Teaching and Research Laboratory of Clinical Analysis at the Universidade Estadual de Maringá, from January 2015 through December 2017. The scheme of this study is described in **Figure 6**. In 707 (69.3%) patients, onychomycosis was confirmed through at least one laboratory test; 417 (58.5%) of the patients tested positive only in the mycological examination, and of these, the positive tests were 290 (41.5%) complemented by positive cultures, with a total of 301 fungal samples. Of these, 51.5% were dermatophytes, 36.2% yeasts, and 12.3% NDM, belonging to the species listed in **Table 2**. Adults aged 30–59 years comprised 54% of the cases, followed by patients aged 60 years or more. The majority of the patients were female, comprising 73.4% of the cases of onychomycosis. The toenails were most often affected, comprising 75.5% of the onychomycosis cases (**Table 3**). Of the patients who accepted to be treated with 10% PE exclusively, a distal lateral lesion was the most common clinical manifestation (**Table 4**).

**Table 2:** Distribution of 301 fungal isolates from cases of onychomycosis confirmed by different cultures in patients seen in the Teaching and Research Laboratory of Clinical Analysis at the Universidade Estadual de Maringá from January 2015 through December 2017.

Year	Dermatophytes		Non-dermatophytes		Total
	*T. rubrum*	*Trichophyton* spp.	NDM	Yeasts	
2015	45 (38.2%)	16 (13.6%)	12 (10.1%)	45 (38.1%)	118 (100%)
2016	35 (39.3%)	10 (11.2%)	13 (14.6%)	31 (34.9%)	89 (100%)
2017	38 (40.4%)	11 (11.7%)	12 (12.8%)	33 (35.1%)	94 (100%)
Total	118 (39.2%)	37 (12.3%)	37 (12.3%)	109 (36.2%)	301 (100%)

*NDM: Non-dermatophytes molds.*

**Table 3:** Demographic data for 707 patients seen at the Teaching and Research Laboratory of Clinical Analysis at the Universidade Estadual de Maringá from January 2015 through December 2017, with laboratory and clinically confirmed onychomycosis.

Year		2015	2016	2017	Total
Sex	Female	199 (74.8%)	160 (73.1%)	160 (71.7%)	519 (73.4%)
	Male	57 (25.2%)	58 (26.9%)	63 (28.3%)	188 (26.6%)
Age	0–14 years	6 (2.3%)	8 (3.7%)	8 (3.5%)	22 (3.1%)
	15–29 years	13 (4.9%)	23 (10.5%)	14 (6.3%)	50 (7.1%)
	30–59 years	153 (57.5)	111 (51.0%)	118 (53.0%)	382 (54.0%)
	60 years or more	94 (35.3%)	76 (34.8%)	83 (37.2%)	253 (35.8%)
Site	Foot	212 (79,7%)	160 (73.4%)	162 (72.6%)	534 (75.5%)
	Hand	54 (20.1%)	58 (26.6%)	61 (27.35)	173 (24.5%)
Total cases of onychomycosis^∗^		266 (100%)	218 (100%)	223 (100%)	707 (100%)

*Total cases of onychomycosis^∗^: confirmed by direct mycological examination and/or culture.*

**Table 4:** Partial results of topical treatment with 10% ethanolic propolis extract of 16 patients over 50 years old with onychomycosis, after 6 months.

Patient sex	Agent	Clinical aspects^∗^			Outcome 6 months of treatment^∗∗^		
		Distal lateral	Proximal	Paroniquia	Complete cure	Partial improvement	Without improvement
F	*T. rubrum*	X			X		
F	*T. rubrum*	X			X		
M	*T. rubrum*	X			X		
M	*T. rubrum*	X			X		
M	*T. rubrum*		X			X	
F	*T. rubrum*	X					X
M	*T. rubrum*	X					X
M	*T. mentagrophytes*	X			X		
F	*T. mentagrophytes*	X				X	
M	*T. verrucosum*		X		X		
M	*T. verrucosum*		X			X	
F	*T. interdigitale*	X			X		
F	*F. solani*	X		X		X	
F	*C. tropicalis*		X	X		X	
F	*C. parapsilosis*		X	X	X		
F	*C. parapsilosis*		X	X	X		

*Clinical aspects^∗^ – The clinical classification was according to Baswan et al., 2017. Outcome 6 months of treatment^∗∗^: Complete cure the nail was fully recovered to its pre-infection appearance and no fungi could be observed in the sample; Partial improvement the ungual lesion was at least 50% smaller and only sparse fungi were present in the sample; Without improvement the appearance and size of the lesions remained similar to the state before the treatment began and abundant fungi were still present in the sample.*

### Treatment

**FIGURE 7:**
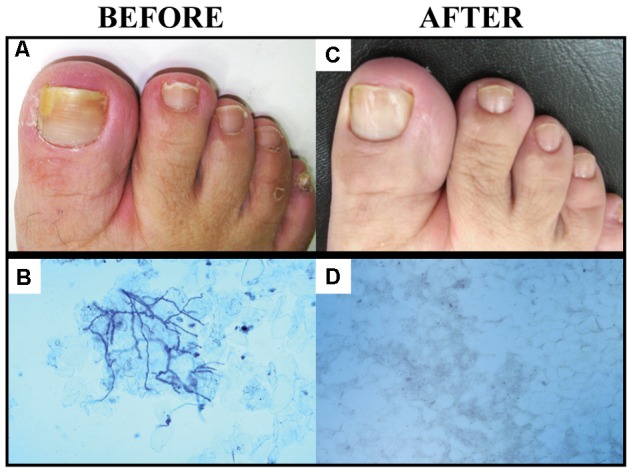
Images showing patients with onychomycosis caused by *Trichophyton* spp. before and after treatment with PE. Patient with toenail onychomycosis before treatment with PE **(A)**; nail scrapings cleared with KOH plus Parker’s blue–black permanent ink, observed by direct microscopy **(B)**; complete resolution of the nail, fully recovered to its initial appearance **(C)**; and no fungi present in the sample **(D)**.

Of the group of 290 patients with laboratory and clinically diagnosed onychomycosis in the present study, 16 gave their written consent for the treatment of onychomycosis exclusively by topical application of 10% PE (**Figure 6**). These 16 cases had between one and eight nails affected, with mild-to-moderate toenail onychomycosis, up to approximately 60% involvement of the nail. After 6 months of treatment, 56.25% showed a complete cure, with total recovery of the initial appearance of the nail and absence of fungi in the sample. Partial or no improvement occurred in 31.25 and 12.5%, respectively. **Table 4** lists the fungal species involved, with the clinical aspects and outcome of each case. **Figure 7** illustrates the clinical appearance of a toenail before and after the treatment.

## Discussion

In order to investigate the antifungal property of PE used for topical treatment of onychomycosis, we conducted studies from the bench to the clinic. Our results demonstrated the possible fungicidal effect of PE and elucidated how the fungus penetrates into the nail. We report successful experiences with the use of 10% PE in the topical treatment of onychomycosis.

Both PEs evaluated in this study were obtained with the Brazilian green propolis from the state of Paraná. The 10% PE used for the treatment was purchased in the commercial pharmaceutical form, manufactured in accordance with the internal quality control of the company. The experimental assays used 30% PE, with the physical and chemical evaluation parameters shown in **Table 1**. Based on these assays, the extracts used in this study showed good physical and chemical characteristics and low cytotoxicity (**Figure 2**). These properties were expected, since the ethanol extracts prepared from this type of propolis were standardized and chemically characterized in previous studies (Bruschi et al., 2003; Dalben-Dota et al., 2010; Tobaldini-Valerio et al., 2016).

The main components of PE are cinnamic acids (mainly compounds with prenyl groups), terpenoid compounds (e.g., sesqui-, di-, and pentacyclic triterpenoids), artepillin C, and phenolic substances (Dota et al., 2011). The active substances in PE are chiefly the phenolic substances, which are responsible for the anti-inflammatory, antimicrobial, and in particular antifungal activity of propolis (Cushnie and Lamb, 2005; Sommez et al., 2005). Several studies have examined the antifungal effects of propolis (Silici and Koc, 2006; Dota et al., 2011; Capoci et al., 2015; Firdaus et al., 2016; Tobaldini-Valerio et al., 2016; Waller et al., 2017), including on the fungi that cause onychomycosis (Oliveira et al., 2006; Galletti et al., 2016). However, little has been published on dermatophyte fungi, which are the main etiological agents of onychomycosis and are refractory to conventional treatment (Siqueira et al., 2009; Agüero et al., 2014).

The difficulty in treating onychomycosis has been related, at least in part, to the organization of filamentous fungi in biofilms. Biofilms are sessile polymicrobial communities surrounded by extracellular polymeric substances (EPS) that confer resistance to treatment and may act as a persistent source of infection, possibly accounting for the resistance to antifungal agents in onychomycosis (Gupta et al., 2016). This property could explain the chronic nature and the difficulty of managing and curing onychomycosis. Besides, the strong adherence of fungi to the nails and the possibility that more than one species of fungus may be involved in onychomycosis reinforce this supposition, because they are normal characteristics of biofilms. Other investigators have suggested that biofilm organization may be related to the persistence of infection and resistance to therapy and long-term cure of onychomycosis (Costa-Orlandi et al., 2014; Toukabri et al., 2017). Interestingly, the activity of propolis against fungal biofilms was previously shown (Capoci et al., 2015; Galletti et al., 2016; Tobaldini-Valerio et al., 2016).

Koc et al. (2005) assayed the *in vitro* antifungal activity of propolis on species of *Trichophyton.* These investigators found that PEs with low MIC values were able to inhibit these dermatophytes, based on this antifungal activity; they suggested that this compound be investigated as a potential antifungal agent for treatment of dermatophytosis. This suggestion, allied to our experience with the antifungal activity of PE (Oliveira et al., 2006; Dota et al., 2011; Capoci et al., 2015; Galletti et al., 2016; Tobaldini-Valerio et al., 2016), motivated our group to conduct more-detailed evaluations of the application of PE to topical treatment of onychomycosis.

As far as we know, no data are available that show the activity of propolis against *Trichophyton* biofilm. In this study, we demonstrated that PE was able to significantly reduce the biofilm formed *in vitro* by *T. rubrum* and *T. interdigitale*, both in total biomass and in the number of viable cells (**Figure 3**). Costa-Orlandi et al. (2014) described the characteristics of the biofilm produced by *Trichophyton* spp., showing that a mature biofilm is composed of a network of hyphae that grow in all directions and are embedded in EPS. These biofilm characteristics explain the difficulty of treating nails with antifungals and reinforce our idea that PE may be efficient for the topical treatment of onychomycosis, mostly after observing the efficiency of PE in reducing a pre-established *Trichophyton* biofilm.

However, it was not yet clear that PE could penetrate the nail and reach its different layers. Recently Gupta et al. (2018) successfully evaluated the penetration of a topical solution of 5% tavaborole through *ex vivo* polished human fingernails, using only visual measurement. However, we chose an *ex vivo* model, which enabled us to show that PAS provided a differential characterization of the spectra of the three components (fungus, nail, and propolis) individually and together, making it possible to determine the capacity of PE (Gregoris and Stevanato, 2010) to permeate or diffuse into the nail (**Figures 4, 5**). PE reached ⅓ of the nail within only 24 h of treatment; i.e., when applied on the dorsal layer, which is the main barrier to the diffusion of medicines, PE penetrated through the nail. Therefore, our results suggest that a repetitive application of PE would rapidly reach all the layers of the nail and provide a successful treatment.

The therapeutic effectiveness of medicines applied topically is related, among other factors, to their diffusion rate (Sugiura et al., 2014). PAS has been applied successfully in the evaluation of the penetration of medicines in non-homogeneous and opaque samples, such as the skin (Ames et al., 2017; Borghi-Pangoni et al., 2017), tooth (Occhi-Alexandre et al., 2018), and nail (Nuglisch et al., 2005). In the present study, PAS efficiently showed that PE was able to reach the deep layers of the nail by itself, which illustrates an additional property of this natural compound. Considering the evidence that filamentous fungi organize in networks and reach the deepest layers of the nail, the challenge is to assure that the topical formulation really reaches all parts of the nail that may be inhabited by the fungi. For this target, Shemer et al. (2016) suggested drilling into the nail as an adjunctive treatment for toenail onychomycosis. According to their conclusion, holes plus topical terbinafine produced a significantly greater improvement in the toenail appearance and higher mycological cure rates compared to treating only the dorsal surface of the nail plate with topical terbinafine. Nevertheless, this is an invasive process that requires a specialized professional.

In the present study, the PE was able to penetrate the nail by itself. Propolis is a compound with a complex chemical composition. During the preparation of PE, the ethanol disperses substances responsible for the biological activity (e.g., phenols), gums, resins, and beeswax (Bruschi et al., 2003, 2006, 2007). This complex mixture results in an adhesive material, which may prolong the time that the formulation is in contact with the application site, increasing the availability of propolis substances to interact with the substrate (Bruschi et al., 2006, 2007; Pereira et al., 2013). The rapid evaporation of ethanol should be also considered. Moreover, the polarity and chemical composition of PE enable good retention and permeation (de Toledo et al., 2015, 2016; Rosseto et al., 2017). Therefore, PE may constitute a good final formulation to be administered to the nail.

The next step in the present study was to evaluate the performance of PE in cases of onychomycosis. Initially, in the epidemiological study, all patients who were seen in the Medical Mycology service of LEPAC during the period evaluated were included in the study. However, for the first phase of the treatment study, only 16 patients who accepted to treat their onychomycosis exclusively with PE were included (**Figure 6**).

Our initial etiological data were in agreement with other epidemiological findings (**Table 2**). *Trichophyton* spp. is the most common genus of dermatophyte isolated from cases of onychomycosis, and the species *T. rubrum* is also the most common in the United States (Bodman and Krishnamurthy, 2017), Germany (Nenoff et al., 2014), and other countries (Segal et al., 2015; Maraki and Mavromanolaki, 2016; Dubljanin et al., 2017).

Demographic data for 707 patients (**Table 3**) indicated that the features of our initial affected population (predominance of females, age above 30 years and toenail) are in accordance with the world literature. Previous studies (Chiacchio et al., 2013; Sigurgeirsson and Baran, 2014; Papini et al., 2015) found a higher frequency of onychomycosis in males. Most of these patients were over 30 years old, as also found by others (Yadav et al., 2015; Kershenovich et al., 2017; Oz et al., 2017). The aging of the skin and its associated structures leads to structural changes that facilitate the entry of microorganisms such as fungi. With progressing age, the nails, mainly the toenails, thicken and suffer traumas even with shoes, providing a favorable environment for the development of these agents.

Particular attention to onychomycosis is needed in the elderly, since this is a growing segment of the population, mostly in developed countries (Hof, 2010). Regardless of the length of life, the elderly person is vulnerable to many diseases and in consequence is usually taking multiple medications, with a high risk of drug interactions, which reinforces the advantages of a topical treatment that is efficient.

There is a consensus that the treatment of onychomycosis still represents a challenge, because of either the few options or the low efficacy of medications available for both systemic and topical treatment. It is essential to search for new options, since in addition to the aesthetic effects, onychomycosis lesions act as an entry point for filamentous fungi that may disseminate and cause invasive infections, which are serious and often fatal (Kim et al., 2016; Kershenovich et al., 2017). This risk is well known for *Fusarium* spp. (Varon et al., 2014) and has also been reported in cases of invasive infections by *T. rubrum* originating from onychomycosis (Talebi-Liasi and Shinohara, 2017).

The results of the current study indicate that PE represents a very promising alternative for treatment of onychomycosis, including cases caused by the genus *Trichophyton.* The cure rate after 6 months of topical treatment (total recovery of the anatomical appearance of the nails and absence of fungi in direct mycological examination) was 56.25% (**Table 4**). A systematic review of topical therapies for toenail onychomycosis showed that FDA-approved topical drugs such as ciclopirox, tavaborole, and efinaconazole produced clinical and mycological cure with reasonable clinical improvement, but with variations according to the treatment plan and combinations used (Gupta et al., 2014). Another, more recent review by the same group (Gupta et al., 2017) showed complete cure with topical treatment using these medicines, at rates from 6–9% for ciclopirox and tavaborole and 15–18% for efinaconazole. The present study found that PE provided a complete cure in 9 of 16 treated patients (56.25%), a higher rate than those obtained with approved medicines. Thus, PE was as efficient as or better than these medications for the treatment of onychomycosis. Tavaborole and efinaconazole, two new topical agents, have demonstrated good nail penetration and high negative culture rates in clinical trials of patients with onychomycosis (Zane et al., 2016); however, they are not available in some countries, including Brazil. If the above rate of complete cures is added to the 31.25% rate of partial cures (patients who are still being treated and will be followed for a longer period of time, 12 months), we could report an 87% index of clinical improvement, a superior performance to the results attained with several other natural products (Halteh et al., 2016) for onychomycosis topical treatment. This efficacy may be related to the cumulative effect of the different properties of propolis, such as antimicrobial, biofilm reducer, anti-inflammatory, and scarring effect (Oryan et al., 2018).

The 16 patients who used only topical PE for their onychomycosis treatment had mild-to-moderate toenail involvement, in concordance with a recent recommendation (Gupta et al., 2017). Data summarized in **Table 4** include the fungal species, clinical aspects of the initial lesion, and the outcome after 6 months of treatment. Of the nine cases with a complete cure, seven of the cases were caused by members of *Trichophyton* and two by *C. parapsilosis*, thus encompassing the common onychomycosis agents and fungal species that are known as difficult to treat (Gupta et al., 2017). Similarly, the five cases with partial cures included a *Fusarium* isolate, another antifungal-resistant microorganism (Galletti et al., 2016). This general picture, despite the small patient population, emphasizes that PE is a very promising topical medication for onychomycosis, as it was effective against all fungal groups involved in onychomycosis (dermatophytes, including anthropophilic species, and non-dermatophytes such as *Fusarium* spp. and *Candida* spp.). Two cases that showed no improvement (12.5%) were caused by *T. rubrum*, a well-known difficult-to-treat onychomycosis agent (Aly et al., 2017). Therefore, PE would be helpful as a sole treatment option, especially in health services without laboratory support. Use of PE would allow patients to start treatment regardless of the species identification, as an empirical treatment, with good chances of success. In addition, topical use of PE allows treatment with a lower risk of drug interaction, especially in elderly patients who use extensive medications (Akhtar et al., 2016). PE would also be useful in children, who have thin, fast-growing nails, facilitating permeation, and treatment (Eichenfield and Friedlander, 2016).

None of our patients treated with PE reported side effects even with long-term use, only some visual discomfort caused by the dark-yellowish color of the treated nail and surrounding skin. This limitation may be explored in future studies. In addition, no one reported an allergic process, even though some allergens have been reported in propolis (Gardana and Simonetti, 2011). This natural product is considered a non-toxic medicine (Capoci et al., 2015). Our present data agree with this statement, since PE induced 50% cell death (**Figure 2**), which is considered an acceptable threshold (Zhao et al., 2012; Guilhelmelli et al., 2016). Assessment of the possible toxic effects of a compound is an essential step in the development of new drugs and therapies, in order to avoid undesired problems during medical interventions (Tyszka-Czochara et al., 2014). It is also important to investigate any harmful effects such as cytotoxicity or a decrease of cell growth in tissues and organs.

Finally, there is concern about the use of over-the-counter and natural remedies for onychomycosis (Halteh et al., 2016), considering the need for standardization and quality control, among other requirements, to produce reliable compounds. These concerns have been allayed for propolis, because the products containing propolis as the active principle are registered, regulated, and approved for use in several pharmaceutical dosage forms in South America (e.g., Brazil and Argentina), the United States, Europe, and Asia (e.g., Japan) (de Toledo et al., 2015, 2016; Rosseto et al., 2017).

Among the limitations of this study are the lack of a placebo group, small sample size, wide variety of pathogens represented, non-randomized population, and lack of stratification by baseline severity or involvement of the nail. However, we note all previous studies were conducted *in vitro*. This is the first study with treated patients but it is not a clinical trial, which should be performed soon considering the promising results found here, from the bench to the patient. Regarding the two concentrations of PE used, for the laboratory studies we preferred to employ the same concentration used in previous studies (30% PE). For the treatment study, we prescribed a commercial product, which was produced according to Brazilian regulations (National Health Surveillance Agency, RDC number 24/2011, and technical note), i.e., 10% PE.

## Conclusion

The findings of the present study suggest that the ethanol PE is a promising natural compound for onychomycosis treatment. Our results confirmed the *in vitro* antifungal activity of PE, against both the planktonic cells and the biofilm formed by species of *Trichophyton*, which are the most common agents of onychomycosis and are usually resistant to conventional antifungals. Furthermore, PE was able to penetrate through the nail by itself, a good property for topical medications, and was not cytotoxic to the cell lines tested. Patients with onychomycosis treated topically with PE twice a day showed excellent clinical improvement within 6 months. Therefore, the extract of propolis is a potential new therapeutic agent for the topical treatment of *Trichophyton* onychomycosis.

## Ethics Statement

All procedures performed in studies involving human participants were in accordance with the ethical standards of the Research Ethics Committee of Universidade Estadual de Maringá, approved with judgment numbers 615.643/2014 and 44.896515.5.0000.0104, with the 1964 Helsinki’s Declaration and its later amendments or comparable ethical standards.

## Author Contributions

FV performed the material collection of nail material, identification of fungi, minimum inhibitory concentration (MIC), anti-biofilm activity, and Photoacoustic Spectroscopy (PAS) experiments. MG and MS performed the collection of nail material, identification of fungi, and MIC. MC performed the translation of the manuscript and data analysis. BK performed the cytotoxicity assays and statistical analysis. LC-H, FS, and MBa conducted the PAS experiments and analysis. MV and VV performed the sequencing of the fungi. VV-P was the dermatologist responsible for the follow-up with the patients. MBr performed the extraction and the characterization of propolis. MN and TS designed and formulated the study and guided the biological tests and the manuscript, as well as its correction. All authors have contributed to the research, writing, and approval of the final version of the manuscript.

## Conflict of Interest Statement

The authors declare that the research was conducted in the absence of any commercial or financial relationships that could be construed as a potential conflict of interest.

## Footnotes

1 http://www.westerdijkinstitute.nl/Collections
